# Supplementary material for: From skylight input to behavioural output: A computational model of the insect polarised light compass
Source: PLoS Comput Biol. 2019 Jul 18;15(7):e1007123. doi: 10.1371/journal.pcbi.1007123 (PMC6638774; doi:10.1371/journal.pcbi.1007123)
Supplement: S1 Table — ID (i), the identity of the POL unit referring to the figures of the main text; θi, the zenith distance of the ith unit; ϕi, the azimuth of the ith unit. The orientation of each unit is a function of its relative azimuth to the heading direction of the dome: αi = ϕi − 90°. (PDF) [file pcbi.1007123.s008.pdf]

Table. Spherical coordinates of the exact positions of the POL units on the dome.

ID (i)	θ^i	ϕ^i	ID (i)	θ^i	ϕ^i	ID (i)	θ^i	ϕ^i
1	5.13	45.00	21	15.39	−105.00	41	25.65	9.00
2	5.13	135.00	22	15.39	−75.00	42	25.65	27.00
3	5.13	−135.00	23	15.39	−45.00	43	25.65	45.00
4	5.13	−45.00	24	15.39	−15.00	44	25.65	63.00
5	10.26	22.50	25	20.52	11.25	45	25.65	81.00
6	10.26	67.50	26	20.52	33.75	46	25.65	99.00
7	10.26	112.50	27	20.52	56.25	47	25.65	117.00
8	10.26	157.50	28	20.52	78.75	48	25.65	135.00
9	10.26	−157.50	29	20.52	101.25	49	25.65	153.00
10	10.26	−112.50	30	20.52	123.75	50	25.65	171.00
11	10.26	−67.50	31	20.52	146.25	51	25.65	−171.00
12	10.26	−22.50	32	20.52	168.75	52	25.65	−153.00
13	15.39	15.00	33	20.52	−168.75	53	25.65	−135.00
14	15.39	45.00	34	20.52	−146.25	54	25.65	−117.00
15	15.39	75.00	35	20.52	−123.75	55	25.65	−99.00
16	15.39	105.00	36	20.52	−101.25	56	25.65	−81.00
17	15.39	135.00	37	20.52	−78.75	57	25.65	−63.00
18	15.39	165.00	38	20.52	−56.25	58	25.65	−45.00
19	15.39	−165.00	39	20.52	−33.75	59	25.65	−27.00
20	15.39	−135.00	40	20.52	−11.25	60	25.65	−9.00

ID (i), the identity of the POL unit referring to the figures of the main text; θ^i, the zenith distance of the ith unit; ϕ^i, the azimuth of the ith unit.
